# Sentinels of synthetics – a comparison of phthalate exposure between common bottlenose dolphins (*Tursiops truncatus*) and human reference populations

**DOI:** 10.1371/journal.pone.0240506

**Published:** 2020-10-15

**Authors:** Leslie B. Hart, Miranda K. Dziobak, Emily C. Pisarski, Edward F. Wirth, Randall S. Wells

**Affiliations:** 1 Department of Health and Human Performance, College of Charleston, Charleston, SC, United States of America; 2 Environmental and Sustainability Studies Graduate Program, College of Charleston, Charleston, SC, United States of America; 3 CSS Inc., NOAA NCCOS Charleston Lab, Charleston, SC, United States of America; 4 National Oceanic and Atmospheric Administration, NOAA NCCOS Charleston Lab, Charleston, SC, United States of America; 5 Chicago Zoological Society’s Sarasota Dolphin Research Program, c/o Mote Marine Laboratory, Sarasota, FL, United States of America; Institute of Deep-sea Science and Engineering, Chinese Academy of Sciences, CHINA

## Abstract

Phthalates are chemical esters used as additives in common consumer goods, such as plastics, household cleaners, and personal care products. Phthalates are not chemically bound to the items to which they are added and can easily leach into the surrounding environment. Anthropogenic drivers, such as coastal plastic pollution and wastewater runoff, increase the exposure potential for coastal marine fauna. Phthalate exposure in free-ranging bottlenose dolphins has been the focus of recent study, with indications of heightened exposure to certain phthalate compounds. The objective of this study was to compare urinary phthalate metabolite concentrations among bottlenose dolphins (*Tursiops truncatus*) sampled in Sarasota Bay, FL, to levels reported in human samples collected as part of the Centers for Disease Control and Prevention’s (CDC) National Health and Nutrition Examination Survey (NHANES). Monoethyl phthalate (MEP) and mono-(2-ethylhexyl) phthalate (MEHP) were the most prevalent metabolites detected in dolphin urine (n = 51; MEP = 29.41%; MEHP = 54.90%). The geometric mean (GM) concentration of MEP was significantly lower for dolphins (GM = 4.51 ng/mL; 95% CI: 2.77–7.34 ng/mL) compared to humans (p<0.05), while dolphin concentrations of MEHP (GM = 4.57 ng/mL; 95% CI: 2.37–8.80 ng/mL) were significantly higher than levels reported in NHANES (p<0.05). Health impacts to bottlenose dolphins resulting from elevated exposure to the MEHP parent compound (diethyl-2-ethylhexyl phthalate, DEHP) are currently unknown. However, given the evidence of endocrine disruption, reproductive impairment, and abnormal development in humans, pursuing investigations of potential health effects in exposed bottlenose dolphins would be warranted.

## Introduction

Phthalates are a class of manmade chemicals added to plastics, personal care products, cleaning solutions, cosmetics, and pesticides to enhance various properties such as lubrication, flexibility, and fragrance [1–3]. These chemicals are concerning to public and wildlife health because of their ubiquitous use in common goods and their potential for endocrine disruption [4–6]. Endocrine disrupting chemicals (EDCs) interfere with the normal production, secretion, or transport of hormones through the body by either mimicking naturally produced compounds or interfering with hormone receptors [7]. Ultimately, this hormone disruption can impact reproduction, development, and/or growth [4, 5, 8–12]. Humans are exposed to phthalates intravenously through the use of medical tubing or via dermal absorption, inhalation, or ingestion resulting from the use of products and materials containing phthalates [13–15]. Because of the environmental ubiquity of phthalates, humans seem particularly at risk of adverse health impacts resulting from chronic exposure.

The Centers for Disease Control and Prevention’s (CDC) National Health and Nutrition Examination Survey (NHANES) is a health assessment of randomly selected individuals across the United States. NHANES data are collected annually and use surveys and specimen collection to evaluate markers of behavioral, mental, physical, and biological health. Blood, urine, and fecal samples are collected to evaluate exposure to environmental chemicals, including phthalate metabolites [16]. Urine is the preferred sampling matrix to evaluate exposure due to the rapid hydrolysis and conjugation of diester phthalate parent compounds, thereby resulting in detectable monoester metabolites [17–20]. Phthalate exposure has been assessed in NHANES urine samples since 1999 [16], and these national surveys often serve as a comparison for studies investigating populations with heightened exposure and risk of adverse health impacts [21–23].

In contrast to persistent organic pollutants (POPs) such as polychlorinated biphenyls (PCBs), dioxins, and furans, phthalates are not considered persistent environmental contaminants, but ongoing release of phthalates into the environment may lead to a chronic exposure risk to wildlife. Marine and aquatic organisms may be exposed through inhalation or ingestion of phthalate-contaminated air, water, sediment, and prey, as well as ingestion of plastic [24–27]. Studies of phthalate exposure have been widespread among marine and aquatic fauna, including the harbor porpoise (*Phocoena phocoena*; [28]), fin whale (*Balaenoptera physalus*; [26, 29]), Risso’s dolphin (*Grampus griseus*; [29]), striped dolphin (*Stenella coeruleoalba*; [29]), common bottlenose dolphin (*Tursiops truncatus*) [29, 30], Atlantic bluefin tuna (*Thunnus thynnus*; [31]), basking shark (*Cetorhinus maximus*; [26]), American alligator (*Alligator mississippiensis*; [32]), European eel (*Anguilla anguilla*; [33]), as well as crustacean, mollusc, and fish species [27, 34]. These studies have detected phthalate parent compounds and/or metabolites in a variety of matrices (e.g., skin, blubber, muscle, urine), with variable concentrations reported within and across species (Table 1).

**Table 1:** Mean concentrations (solid mass reported as wet weight (w.w.) or dry weight (d.w.)) of monoethyl phthalate (MEP) and mono-(2-ethylhexyl) phthalate (MEHP) with corresponding ranges and standard deviations as reported in varying matrices from other marine species.

Species	N	Sampling Pd.	MEP mean (s.d.)	MEP range	MEHP mean (s.d.)	MEHP range	Matrix
Harbor porpoise (*Phocoena phocoena*; [28])	100	2016–2017	5.99 ng/g w.w.	2.62–17.4 ng/g w.w.	-	-	Liver
Fin whale (*Balaenoptera physalus*; [26])	5	2007–2013	-	-	Approximately 190 ng/g l.b. reported graphically	-	Blubber
Fin whale (*Balaenoptera physalus*; [29])	3	2014	-	-	<LOD	-	Blubber
Risso’s dolphin (*Grampus griseus*; [29])	1	2014	-	-	463.7 ng/g d.w.	-	Blubber
Common Bottlenose dolphin (*Tursiops truncatus*; [29])	1	2014	-	-	1770 ng/g d.w.	-	Blubber
Striped dolphin (*Stenella coeruleoalba*; [29])	2	2014	-	-	1720 ng/g d.w.	-	Blubber
Atlantic Bluefin tuna (*Thunnus thynnus*; [31])	23	2012	-	-	2.13 (1.52) ng/g w.w.	1.58–6.30 ng/g w.w.	Muscle
Basking shark (*Cetorhinus maximus*; [26])	6	2006–2013	-	-	Approximately 90 ng/g l.b. reported graphically		Muscle
American alligator (*Alligator mississippiensis*; [32]) Everglades	9	Sampling year not reported	-	-	4,540 (11,800) ng/mL	ND-35,700 ng/mL	Urine
American alligator (*Alligator mississippiensis*; [32]) Okeechobee–Belle Glade	10	Sampling year not reported	-	-	1,490 (1,290) ng/mL	ND-11,500 ng/mL	Urine
American alligator (*Alligator mississippiensis*; [32]) Okeechobee–Moonshine Bay	10	Sampling year not reported	-	-	1,290 (3,470) ng/mL	ND-11,100 ng/mL	Urine
American alligator (*Alligator mississippiensis*; [32]) Woodruff	9	Sampling year not reported	-	-	56.4 ng/mL	ND-506 ng/mL	Urine
American alligator (*Alligator mississippiensis*; [32]) Apopka	12	Sampling year not reported	-	-	-	-	Urine
European eels (*Anguilla Anguilla*; [33])	117	2010	33 (108) ng/g d.w.	-	282 (206) ng/g d.w	-	Fillet muscle
Roach (*Rutilus rutilus*; [34])	4	Sampling year not reported	53 (15.3) ng/mL	-	15.5 ng/mL	-	Bile
Roach (*Rutilus rutilus*; [34])	4	Sampling year not reported	28.6 (4.9) ng/mL	-	122 (7.7) ng/mL	-	Plasma
Roach (*Rutilus rutilus*; [34])	4	Sampling year not reported	263 (154) ng/g d.w.	-	237 (81) ng/g d.w.	-	Liver
Prawn [27]	20	2013	ND-6.82 ng/g w.w.	-	ND-61.6 ng/g w.w.	-	Edible tissue
Mollusc [27]	6	2013	0.42–3.31 ng/g w.w.	-	7.50–11.6 ng/g w.w.	-	Edible tissue
Fish [27]	69	2013	0.06–4.70 ng/g w.w.	-	ND-24.8 ng/g w.w.	-	Edible tissue

A recent study of free-ranging common bottlenose dolphins sampled in Sarasota Bay, Florida, USA during 2016 and 2017 (n = 17) detected phthalate metabolite concentrations among 70% of dolphins sampled, suggesting prevalent environmental exposure to these man-made chemicals [30]. As top-level predators with a long lifespan (>60 years; [35]), year-round resident bottlenose dolphins serve as sensitive gauges to detect disturbances in their local environment [36]. This has been demonstrated in epidemiologic studies of dolphin health impacts linked with PCBs [37, 38], harmful algal blooms [39], and oil-associated toxin exposure [38, 40]. Unfortunately, the extent, sources, and impacts of widespread phthalate exposure in bottlenose dolphins are not yet understood. The objective of this study was to compare bottlenose dolphin phthalate metabolite concentrations to levels reported for human reference populations (i.e., NHANES) and use a One Health approach to develop hypotheses for phthalate-associated health impacts for exposed bottlenose dolphins. “One Health” is a term used to describe gained knowledge of wildlife health by studying humans, and vice versa [41]. In this study, phthalate-associated health effects reported in human epidemiological studies were used to predict potential dolphin health impacts that will be explored in future investigations.

## Materials and methods

### Dolphin study population

Bottlenose dolphins have been the subject of population and health studies in Sarasota Bay, FL since 1970 [42]. The study area includes inshore waters between southern Tampa Bay (approximately 27.56°N) and Venice Inlet (approximately 27.10°N), and offshore of the barrier islands to approximately 82.75°W. Using well-established techniques developed and refined over 50 years, free-ranging dolphins were encircled by a net and temporarily restrained to collect biological samples indicative of an individual’s health [36]. The sex of each dolphin was determined by physical examination, and age was estimated by either known birth year or the observation of dentinal growth layers [43]. A combination of factors were used to determine maturity status including age (≥ 10 years; [44]), calving history, pregnancy diagnosis via ultrasonography, or sex hormone concentrations [45, 46]. Bottlenose dolphin health assessments were conducted under scientific research permit #522–1785, #15543, and #20455 from the National Oceanic and Atmospheric Administration’s (NOAA) National Marine Fisheries Service (NMFS), and research studies were reviewed and approved annually by Mote Marine Laboratory’s Institutional Animal Care and Use Committee (IACUC).

### Urinary phthalate metabolite detection and quantification

This study relied upon bottlenose dolphin urinary metabolite concentrations reported in Hart et al. [30] (sample years 2016–2017), as well as results from analyses conducted on samples collected 2010–2015 and 2018–2019. Urine samples were opportunistically collected aseptically via catheterization from bottlenose dolphins during routine health assessments conducted under permit from the National Oceanic and Atmospheric Administration’s (NOAA) National Marine Fisheries Service (NMFS) between 2010 and 2019, as described in Wells [42] and Hart et al. [30]. Archived urine samples for the years 2010–2015 were retrieved from the Sarasota Dolphin Research Program’s specimen bank, where urine samples were stored frozen at -80°C. The protocol for sample collection and storage for years 2016–2019 are described in Hart et al. [30]. All urine samples were screened for eight phthalate metabolites including: monomethyl phthalate (MMP), monoethyl phthalate (MEP), monoisobutyl phthalate (MiBP), monobutyl phthalate (MBP), monobenzyl phthalate (MBzP), mono-(2-ethylhexyl) phthalate (MEHP), mono-(2-ethyl-5-oxohexyl) phthalate (MEOHP), and mono-(2-ethyl-5-hydroxyhexyl) phthalate (MEHHP). Methods for analyzing phthalate metabolite concentrations in bottlenose dolphin urine were based on protocols established in Hart et al. [30] and conducted at the NOAA National Centers for Coastal Ocean Science (NCCOS) lab in Charleston, SC, USA. Briefly, urine samples were spiked with ^13^C-labeled internal standards prior to an enzymatic de-glucuronidation step. Phthalate metabolites were isolated by solid phase extraction (SPE) using an Agilent Bond Elute Nexus SPE. Male samples that had excess sperm were centrifuged (1,000 rpm for 10 minutes) prior to extraction to separate the urine and prevent the SPE cartridge from becoming clogged. Analytical separation, detection, and quantification of phthalate metabolites in urine were performed using high performance liquid chromatography (HPLC; Agilent 1100) paired with electrospray ionization (ESI) tandem mass spectrometry (AB Sciex API 4000; ESI- mode). Compounds were separated using a C18 analytical column (Waters XBridge 50 mm x 2.1 mm; 2.5 μm particle size) with a gradient mobile phase of HPLC water with 0.1% acetic acid and acetonitrile with 0.1% acetic acid. Analytes of interest were identified using multiple reaction monitoring (MRM; S2 Table) and quantified against standard calibration curves [17, 30], and the limit of detection (LOD) was determined for each metabolite based on the lowest point of the calibration curve that could be detected on the instrument, divided by the volume of urine extracted. Complete details of the instrumental methodology is detailed in Hart et al. [30]. As reported in Hart et al. [30] quality assurance and control methods included laboratory spikes, laboratory blanks, field blanks, a standard reference material (SRM 3672), and matrix spikes. Both lab and field blanks were used to correct urine samples for equipment contamination. Urine samples were run in batches of 10–12, with one corresponding blank per batch. Field blanks were taken in triplicate for each week where sampling occurred and used to correct all samples in the corresponding year. In addition, calibration verification was conducted for each batch of urine samples to ensure the integrity of the calibration curve.

### Human reference population data

Human geometric mean concentrations (ng/mL) were retrieved from NHANES for the following study periods: 2009–2010; 2011–2012; 2013–2014; 2015–2016; concentrations used for analyses were not corrected for creatinine [16]. These study periods were selected due to the temporal overlap with our bottlenose dolphin samples (2010–2019), although NHANES concentrations were unavailable for years following 2016. Phthalate metabolite concentrations were measured in randomly selected individuals from the total NHANES sample population for a given study period (n~2,500; [16]). Metabolite concentrations were quantified from urine samples using mass spectrometry in the CDC’s Environmental Health Laboratory [17], and non-creatine corrected measurements were used for comparisons to bottlenose dolphins [16].

### Statistical analysis

Minimum, maximum, and geometric mean (GM) concentrations were calculated for all phthalate metabolites where concentrations exceeded the limit of detection (LOD) for at least 10% of the bottlenose dolphin study sample. Geometric means were calculated for all detectable concentrations across the entire study period (2010–2019). Concentrations were natural log-transformed prior to testing for correlation between major metabolites. Geometric mean concentrations of detectable phthalate metabolites (i.e., concentrations >LOD) were compared between bottlenose dolphins and human reference populations by evaluating overlap of the 95% confidence intervals for each pairwise comparison (e.g., dolphin GM (2010–2019) vs. NHANES GM (2009–2010) [47]. For each NHANES study period (2009–2010; 2011–2012; 2013–2014; 2015–2016), comparisons between bottlenose dolphins and humans relied on reported geometric mean concentrations for the total NHANES sample (i.e., all age groups, gender, and race/ethnicity). Statistical significance was evaluated using α = 0.05 and all analyses were performed using Statistica (v. 13.3, Tibco Software Inc., Palo Alto, CA) or R (v. 3.6.1, R Foundation for Statistical Computing, Vienna, Austria) computing software.

## Results

### Study sample details

Between 2010 and 2019, 69 urine samples were screened for detectable concentrations of phthalate metabolites. These samples included 17 dolphin specimens reported in Hart et al. [30]. Thirteen dolphins were repeatedly evaluated in Sarasota Bay health assessments during the study period, but only the most recent specimens were used for the analysis herein (n = 51 unique individuals). More than half of the dolphins were female (58.82%), and 66.67% were considered adults.

### Overall phthalate metabolite detection in sampled bottlenose dolphins

Detectable concentrations (i.e., concentrations > LOD) of at least one metabolite were measured in 74.51% of individual dolphins sampled (n = 51; 2010–2019). Limits of detection are provided in Table 2. In addition to data reported by Hart et al. [30], the most commonly detected metabolites were MEHP (n = 28) and MEP (n = 15; S1 Table).

**Table 2:** Range of limits of detection for phthalate metabolites (ng/mL) measured in urine samples from common bottlenose dolphins sampled during health assessments conducted in Sarasota Bay, FL, USA (2010–2019).

	MMP	MEP	MEHP	MEOHP	MEHHP	MBzP	MBP	MiBP
**LOD**	0.10–0.167	1.0–3.43	0.24–0.60	0.10–0.70	0.20–0.90	0.10–0.80	0.50–0.85	0.50–0.983

This table includes limits of detection reported in Hart et al. [30].

### Findings by major metabolites

MEHP was the most commonly detected metabolite among sampled bottlenose dolphins (54.90%), with a geometric mean concentration of 4.57 ng/mL (95% CI:2.37–8.80 ng/mL) and overall range of <LOD to 76.60 ng/mL (Table 3). Approximately 29% of screened dolphins had detectable concentrations of MEP, with a geometric mean of 4.51 ng/mL (95% CI: 2.77–7.34 ng/mL) and range of <LOD to 33.40 ng/mL (Table 3). Natural-log transformed concentrations of MEP and MEHP were not significantly correlated (r = -0.12; p = 0.41). In fact, only nine of the 28 dolphins (32.14%) with detectable concentrations of MEHP had detectable concentrations of MEP.

**Table 3:** Summary of common phthalate metabolites (ng/mL) detected in common bottlenose dolphins sampled during health assessments conducted in Sarasota Bay, FL, USA (2010–2019).

	MEP	MEHP
**Number >LOD**	15	28
**% Above Limit of Detection**	29.41	54.90
Minimum^1^	1.30	0.26
Maximum^1^	33.40	76.60
GM among detects (95% CI)^1^	4.51 (2.77–7.34)	4.57 (2.37–8.80)

^1^reported for dolphins with concentrations >LOD for metabolite.

GM = geometric mean.

### Comparisons between bottlenose dolphin and human concentrations

**Fig 1:**
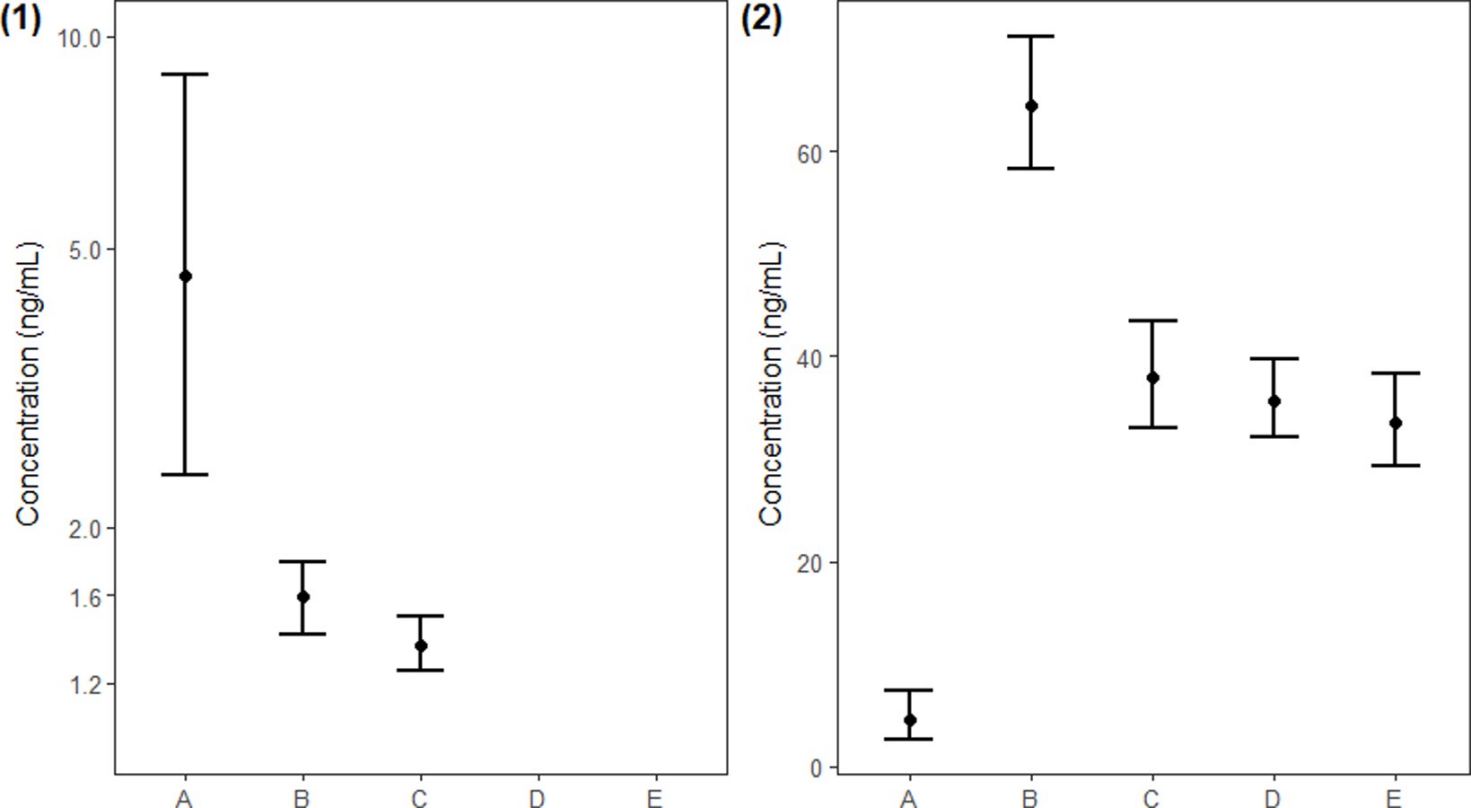
Geometric mean concentrations and 95% confidence intervals for detectable phthalate metabolites for Sarasota Bay bottlenose dolphins (“A”) and NHANES human reference populations (“B-E”: 1) MEHP (n = 28); 2) MEP (n = 15). Bottlenose Dolphin (A); NHANES 2009–2010 (B); NHANES 2011–2012 (C); NHANES 2013–2014 (D); NHANES 2015–2016 (E).

Comparisons to human urinary phthalate metabolite concentrations were conducted for detectable concentrations of MEP and MEHP. Geometric mean concentrations of MEP ranged between 33.60 and 64.40 ng/mL for NHANES study samples (2009–2016; [16]; Table 4). The geometric mean concentration of MEP for bottlenose dolphins (4.51 ng/mL; 95% CI: 2.77–7.34 μg/L) was significantly lower than NHANES for all study years (p<0.05, Table 4 and Fig 1). For MEHP, NHANES geometric mean concentrations were only reported for 2009 (1.59 ng/mL) and 2010 (1.36 ng/mL) because of the high prevalence of non-detectable concentrations in subsequent years ([16]; Table 4). In our study, the geometric mean concentration of detectable MEHP (4.57 ng/mL; 95% CI:2.37–8.80 ng/mL) was significantly higher than NHANES 2009 and 2010 (p<0.05, Table 4 and Fig 1).

**Table 4:** Comparison of detectable concentrations of urinary phthalate metabolites between bottlenose dolphins sampled in Sarasota Bay, FL, USA (2010–2019) and human reference populations (NHANES; 2009–2010, 2011–2012, 2013–2014, 2015–2016).

	Bottlenose Dolphins	NHANES 2009–2010	p^3^	NHANES 2011–2012	p^3^	NHANES 2013–2014	p^3^	NHANES 2015–2016	p^3^
**MEHP**			<0.05		<0.05		**-**		-
* N*	28	2,749		2,489		2,685		2,975	
* GM_detects*^*1*^ *(95% CI)*	4.57 (2.37–8.80)	1.59 (1.41–1.79)		1.36 (1.25–1.49)		NA^2^	-	NA^2^	-
**MEP**									
* N*	15	2,749	<0.05	2,489	<0.05	2,685	<0.05	2,975	<0.05
* GM_detects*^*1*^ *(95% CI)*	4.51 (2.77–7.34)	64.40 (58.30–71.20)		37.90 (33.00–43.50)		35.70 (32.10–39.80)		33.60 (29.30–38.40)	

^1^reported for dolphins with concentrations >LOD for metabolite; concentration is reported in ng/mL

^2^proportion <LOD was too high to calculate reliable estimate [16]

^3^p value evaluated based on 95% confidence interval overlap [47]

## Discussion

This study revealed prevalent exposure (74.51%, n = 51) to phthalates among free-ranging bottlenose dolphins sampled in Sarasota Bay, FL (2010–2019). MEHP was detected in over half of the individuals sampled (n = 28), while MEP was detected in nearly one-third (n = 15) of bottlenose dolphins. Detectable concentrations of these metabolites were highly variable for bottlenose dolphins in this sample (MEHP: 0.39–76.60 ng/mL; MEP: 1.60–33.40 ng/mL), which has been observed in other species (e.g., American alligator, Mediterranean fin whale, harbor porpoise; Table 1), and could be attributed to diet, metabolism, or spatiotemporal exposure differences [25, 32]. Environmental introduction of phthalates can occur via wastewater contamination, groundwater intrusion via landfill leakage, surface runoff, direct application (e.g., agricultural, residential, and industrial pesticides and fertilizers), or atmospheric evaporation [1, 3, 48, 49]. Removal of phthalate metabolites from wastewater is variable, ranging between 29% and 100% depending on the compound, treatment methodology, and treatment time [48, 49]. Once in the environment, persistence in different substrates can be affected by temperature, precipitation, microbial conditions, and chemical properties [49].

### Dolphin-human comparisons: MEP and MEHP

Bottlenose dolphin MEP concentrations were significantly lower than reported exposure in human reference populations [16]. MEP is the monoester metabolite of diethyl phthalate (DEP), which is added to a wide range of commercial goods including plastic items, food packaging, personal care products, cosmetics, pesticides, and some medications [1, 50–52]. Humans are directly exposed via inhalation, ingestion, or dermal absorption of products made with DEP or contaminated by DEP, albeit at variable concentrations [1, 14]. Epidemiologic studies of human exposure to DEP demonstrate higher risk among users of certain personal care products, especially cosmetics and products containing fragrance [53–58]. Given the primary sources for DEP exposure in humans, it is not surprising that bottlenose dolphin concentrations of MEP were significantly lower than NHANES levels. Compared to other commonly used phthalate esters, DEP has a lower molecular weight, is more quickly degraded by microbes, and has a shorter half-life in marine environments (< 1 day to 2 weeks; [49, 59]). As a result, DEP may be less bioavailable than other phthalate parent compounds for free-ranging bottlenose dolphins.

The geometric mean concentration of MEHP for bottlenose dolphins was significantly higher than NHANES concentrations [16]. MEHP is a metabolite of di-(2-ethylhexyl) phthalate (DEHP), which is primarily used to increase the flexibility of plastic (e.g., polyvinyl chloride, food packaging, wire covering, toys, medical tubing; [3, 50, 60]). DEHP can also be found in cosmetics, personal care products, oil and paint [3], although studies have suggested this is due to migration from the plastic containers housing these products [14, 61]. Because of weak bonds with the corresponding substrate, DEHP can be easily leached into food and the environment [3, 60].

Human exposure occurs primarily through the ingestion of dust and food stored in packaging containing DEHP, particularly dairy, meats, and other fatty foods as DEHP is lipophilic [3, 14, 60–62]. The highest doses of exposure, however, occur among people requiring transfusions or dialysis due to DEHP-containing medical equipment [3]. Because DEHP is one of the most abundantly used phthalates, contamination of the natural environment is expected. DEHP introduction into the marine environment can occur via agricultural [63] and non-permeable surface runoff [48], as well as wastewater effluent, although removal can be quite high depending on treatment methods (e.g., up to 95%, [48]). DEHP is the most prevalent phthalate detected in freshwater, soil, atmospheric and landfill leachate samples, and this environmental pervasiveness is likely due to many factors including high production, increased urbanization, and chemical properties that slow down the biodegradation process [49]. Resident dolphins in Sarasota Bay, FL are considered selective feeders, choosing soniferous fish disproportionately relative to their availability [64, 65]. While diet may be a source of exposure to DEHP for bottlenose dolphins, evidence from Staples et al. [59] suggests that prey ingestion is not likely the primary exposure route. In fact, Staples et al. [59] demonstrated that phthalate ester concentrations actually decrease with increasing trophic levels and suggested that higher-order metabolic biotransformation might outpace bioaccumulation.

Plastics are of increasing concern to environmental health because of their ubiquitous use in industrial settings and in the production of many commercial goods [66], combined with the fact that plastic waste materials are slow to degrade and therefore persist in the environment [67]. Geyer et al. [68] estimated a global plastic production of 380 million tons in 2015, of which 11% was polyvinyl chloride. Plastic additives, composed primarily of plasticizers such as phthalates, accounted for 7% of non-fiber plastic mass [68]. Approximately 60% of plastics are disposed of in landfills or the natural environment [68], so it seems reasonable that plastic pollution could be a source of phthalate exposure for marine fauna. Marine plastic debris is often categorized by size [67]; macro- and mesoplastics (≥ 5mm diameter) enter the marine environment directly as waste, while microplastics may not be filtered out by water treatment facilities or result from fragmentation of larger plastic items [24, 67]. Eriksen et al. [69] estimated that our oceans contain over 5.25 trillion plastic particles, of which 92.4% are microplastics. Previous cetacean studies provide evidence of a link between environmental microplastic contamination and phthalate exposure, based on blubber samples of stranded cetaceans and water/plankton samples from nearby seas and estuaries [25, 26, 29]. MEHP concentrations measured in blubber and muscle samples from stranded fin whales and bycaught basking sharks were higher in animals sampled in regions with significantly higher water and plankton microplastic concentrations [25, 26]. While the potential exposure sources of DEHP and other phthalate compounds are not yet understood for Sarasota Bay bottlenose dolphins, the detection of high concentrations of MEHP warrants further investigation.

### Potential implications for bottlenose dolphin exposure and health impacts

Between 2010 and 2019, Sarasota Bay bottlenose dolphin urinary concentrations of MEHP ranged between <LOD and 76.60 μg/L, with a geometric mean concentration of 4.57 μg/L (95% CI: 2.37–8.80 μg/L). Upon exposure, DEHP is hydrolyzed into MEHP and conjugated before urinary excretion. Hydrolysis occurs in the liver through mechanisms involving the alpha-mediated enzymes of the peroxisome proliferator-activated receptor (PPAR⍺) and efficiency can vary among individuals. Metabolites of DEHP (e.g., MEHP, MEOHP, MEHHP) can bind to PPAR⍺ and disrupt normal kidney, liver, heart, and reproductive function [60]. In humans, higher MEHP concentrations have been associated with myriad health impacts including decreased oocyte counts [70], early pregnancy loss [71], reduced sperm quality [72], and abnormal reproductive development [73], among others. These phthalate-associated health impacts may be due to interference in steroid, sex, or thyroid hormone circulation, but the occurrence and magnitude of endocrine disruption seem to vary by phthalate ester type, degree of exposure, sex, pregnancy, and age [3–5, 8–12, 60, 74–79]. While epidemiological studies in humans and experimental studies in laboratory rodents indicate potential health risks for bottlenose dolphins, we found that direct comparisons to human studies were hindered as most papers reported concentrations were adjusted for creatinine or specific gravity. Additionally, NHANES concentrations of MEHP have declined in recent years [16], suggesting reduced exposure and inhibiting studies to identify and validate health impacts. Thus, continued study of Sarasota Bay bottlenose dolphins will enable epidemiological investigations of hormonal, physiological, and reproductive correlates with phthalate exposure, due in large part to long-term monitoring efforts and regularly conducted health assessments.

### Study strengths and limitations

This study relied upon well-established analytical methods developed by the CDC to screen for phthalate metabolites in mammalian urine samples. Urinary phthalate metabolites are considered the most reliable indicators of exposure in human populations due to the rapid metabolism of these chemicals, and because sampling equipment can be contaminated with parent compounds during analysis [18, 80, 81]. Additionally, several methods were used to avoid sample and analytical contamination and validate reported phthalate metabolite data measured in these dolphin samples. The cross-sectional study design, however, reflects prevalent, rather than cumulative, exposure. Studies in humans suggest urinary phthalate metabolite concentrations measured in spot urine samples can reflect prior exposure to parent compounds of a time spanning months to a year (3–6 months; [17, 82]); however, this information is not yet available for dolphins.

To our knowledge, this is the largest assessment of phthalate exposure within any wild marine mammal species. The large sample size provided statistical power to facilitate comparisons between dolphin and human concentrations. To facilitate comparability between study samples, we selected NHANES years that overlapped the timing of our dolphin sample collection, thereby helping to control for decreasing trends in human exposure to MEP and MEHP over time [16]. We observed significant differences in exposure to MEP (lower in dolphins) and MEHP (higher in dolphins), but despite having different routes and sources for exposure, metabolic differences between dolphins and humans should also be considered as a possible explanation for divergent metabolite concentrations. Resting metabolic rate (RMR) for a 150 kg bottlenose dolphin in Sarasota Bay is estimated to be 3.9 mL O_2_ min^-1^ kg^-1^ [83], while McMurray et al. (2014) [84] estimate an average RMR between 2.8 mL O_2_ min^-1^ kg^-1^ and 3.0 mL O_2_ min^-1^ kg^-1^ for men and women. Physiologic adaptations to help dolphins dive, thermoregulate, and forage are likely to impact the biotransformation of phthalate parent compounds; however, these mechanisms, as they relate to phthalate metabolism, are not currently understood.

Finally, laboratory and human epidemiological studies investigating phthalate-related health impacts have demonstrated mixed findings [3, 72, 85] likely due to biases in study design, data collection, or sampling demographic. As such, future studies to investigate bottlenose dolphin phthalate exposure and health impacts should consider the influence of potential confounding variables.

## Conclusions

Findings from this study indicate higher exposure to MEHP among bottlenose dolphins inhabiting Sarasota Bay, FL, compared to U.S. human reference populations; however, the significance of these results is uncertain. For decades, bottlenose dolphins have been considered sentinels of environmental health and indicators of potential health risks to human users of coastal resources [86]. For example, studies of bottlenose dolphins inhabiting waters near Brunswick, GA and Miami, FL, documented unprecedented exposure to toxic, polychlorinated biphenyl compounds (PCBs; [87–90]), which corresponded with high exposure risks for humans living in the same area [91]. Backer et al. [91] suggest that because of trophic concurrence, dolphins can gauge and even predict environmental pollution risks for coastal human populations (and vice versa). Similarly, Rabinowitz et al. [92] state that ‘shared health risks’ can facilitate the comparison of exposure risks and corresponding health outcomes between wildlife and human populations. Health impacts to bottlenose dolphins resulting from elevated exposure to the MEHP parent compound (DEHP) are currently unknown. Accordingly, studies relying on long-term reproductive health data collected from Sarasota Bay bottlenose dolphins to investigate associations between phthalate exposure and indicators of endocrine disruption, reproductive impairment, or abnormal growth and development are underway.

## Supporting information

S1 Table Detectable concentrations of MEP and MEHP (ng/mL) among common bottlenose dolphins (*Tursiops truncatus*) sampled in Sarasota Bay, FL, USA (2010–2019). (DOCX)

S2 Table List of phthalate monoester metabolite MRM transitions used for identification and quantitation. (DOCX)
