# Supplementary material for: Sentinels of synthetics – a comparison of phthalate exposure between common bottlenose dolphins (Tursiops truncatus) and human reference populations
Source: PLoS One. 2020 Oct 15;15(10):e0240506. doi: 10.1371/journal.pone.0240506 (PMC7561143; doi:10.1371/journal.pone.0240506)
Supplement: S1 Table — (DOCX) [file pone.0240506.s001.docx]

**S1 Table**

| **Individual** | **MEP Concentration (ng/mL)** | **MEHP Concentration (ng/mL)** |
| --- | --- | --- |
| 1 | 4.35 | 30.29 |
| 2 | 3.10 | 1.70 |
| 3 | 8.66 | 1.95 |
| 4 | 2.42 | 1.90 |
| 5 | 6.31 | 37.00 |
| 6 | 4.68 | 0.39 |
| 7 | 3.40 | 0.55 |
| 8 | 33.40 | 2.00 |
| 9 | 21.20 | 1.50 |
| 10 | 5.30 | ≤LOD |
| 11 | 2.85 | ≤LOD |
| 12 | 1.30 | ≤LOD |
| 13 | 2.44 | ≤LOD |
| 14 | 4.23 | ≤LOD |
| 15 | 1.60 | ≤LOD |
| 16 | ≤LOD | 2.10 |
| 17 | ≤LOD | 27.10 |
| 18 | ≤LOD | 2.19 |
| 19 | ≤LOD | 0.90 |
| 20 | ≤LOD | 36.90 |
| 21 | ≤LOD | 49.20 |
| 22 | ≤LOD | 28.12 |
| 23 | ≤LOD | 1.00 |
| 24 | ≤LOD | 31.10 |
| 25 | ≤LOD | 76.60 |
| 26 | ≤LOD | 15.99 |
| 27 | ≤LOD | 28.40 |
| 28 | ≤LOD | 0.26 |
| 29 | ≤LOD | 5.90 |
| 30 | ≤LOD | 3.91 |
| 31 | ≤LOD | 2.31 |
| 32 | ≤LOD | 6.35 |
| 33 | ≤LOD | 3.10 |
| 34 | ≤LOD | 0.39 |
