# Supplementary material for: Sentinels of synthetics – a comparison of phthalate exposure between common bottlenose dolphins (Tursiops truncatus) and human reference populations
Source: PLoS One. 2020 Oct 15;15(10):e0240506. doi: 10.1371/journal.pone.0240506 (PMC7561143; doi:10.1371/journal.pone.0240506)
Supplement: S2 Table — (DOCX) [file pone.0240506.s002.docx]

**S2 Table**

| Analyte | Ion transition  parent→daughter (m/z) |
| --- | --- |
| ^13^C_4_-MEP | 197 → 79 |
| MMP | 179 → 77 |
| MEP | 193 → 77 |
| ^13^C_4_-4-MeUMB | 179 → 135 |
| 4-MeUMB | 175 → 133 |
| ^13^C_4_-MEHHP | 297 → 145 |
| MiBP | 221 → 77 |
| MBP | 221 → 77 |
| MEHHP | 293 → 145 |
| MEOHP | 291 → 121 |
| ^13^C_4_-MBzP | 259 → 186 |
| MBzP | 255 → 183 |
| ^13^C_4_-MEHP | 281 → 137 |
| MEHP | 277 → 134 |
